# Patterns of Tooth Extraction at a Student Dental Clinic in Palestine

**DOI:** 10.7759/cureus.46614

**Published:** 2023-10-07

**Authors:** Mohammad Assaf, Kareem Abu Libdeh

**Affiliations:** 1 Faculty of Dentistry, Al-Quds University, Jerusalem, PSE

**Keywords:** edentulism, incidence tooth loss, dentistry, student clinics, tooth extraction

## Abstract

Introduction: While the ideal approach in dentistry is to preserve teeth and restore their functionality within the oral cavity, there are circumstances where tooth extraction becomes a necessary and routine dental procedure. In cases where preserving the tooth is not feasible due to unavoidable reasons, extraction may be the preferred choice to ensure the attainment of stable oral health. So, the present study aims to elaborate on the trends in extraction in student dental clinics in Palestine.

Materials and methods: The present study is a cross-sectional study. Informed consent was obtained from the participants before enrolling in the study. Patients from all the departments undergoing extraction procedures were included in the present study. Patient information such as age, sex, and main reason for tooth extraction was collected. A dental record was noted for the number of teeth extracted, the type of the teeth, and the condition of the teeth extracted. All the data collected was entered in the spreadsheet, and frequency was calculated for each variable.

Results: The present study showed that third molars were the most extracted teeth, and dental caries was the primary cause of extraction. In the “clearance cases,” lower canines were the most commonly extracted teeth. In the “non-clearance cases,” lower incisors and lower first molars were the most commonly extracted teeth.

Conclusion: The present study gives an overview of tooth extraction patterns in student dental clinics in Palestine. Further studies are required to evaluate and compare the prevalence and causes of tooth loss in different settings.

## Introduction

Dental extraction is a dento-alveolar surgical therapeutic intervention requiring the removal of fractured, carious, periodontally compromised, or impacted teeth [1]. Although it is best to preserve teeth and restore their function in the oral cavity, tooth extraction is still a routinely performed dental procedure. For some unavoidable reasons, when the tooth cannot be salvaged, the best choice may be to extract it to obtain stable oral health [2]. Oral diseases that can end up with the loss of permanent teeth are caused by various reasons, most of which can be prevented with good maintenance and early detection of the cause [3,4]. Oral infections such as tooth caries and periodontal diseases usually cause most extractions in modern communities [5]. When the disease reaches a certain limit, it is considered untreatable and may be referred to a dental surgeon for extraction [6,7]. The World Health Organization has been aware of the consequences of tooth loss on general health and has declared since 1992 that edentulousness will remain a concern for several decades; a milestone of having at least 20 functioning teeth has been set as a target to accomplish in the future [8].

Al-Quds University (AQU) is one of the two universities that have dental schools in the West Bank, Palestine. Most dental services are provided for free at the student dental clinics at AQU, which mainly attract patients with low socio-economic levels. The dental clinics are located next to the main campus of AQU, which is located in the town of Abu-Dis, Jerusalem. Attendees of these clinics are patients from the nearby Palestinian cities and villages of Jerusalem, Bethlehem, Jericho, and Ramallah. Tooth extraction is a procedure that is performed by dental students in their fourth and fifth dental years. Only complicated cases are rejected at AQU and referred to specialized clinics. Otherwise, a supervisory surgeon accompanies students during the tooth extraction, whether it is a simple or surgical extraction. Such treatments are provided free of charge to attending patients.

Dental clinicians perform dental extractions on a routine basis. The incidence of tooth extraction can vary from one country to another based on the prevalence of dental diseases, dental prophylaxis level, and awareness among the community [9]. Since the cause of the extraction can be varied due to the location, the pattern of the extraction can also be influenced [10]. An Indonesian study reported that tooth loss was associated with factors such as older age, functional disability, tobacco consumption, and low social capital [11]. In another study carried out in Saudi Arabia, dental caries was considered the primary cause of extraction, followed by orthodontic reasons and lastly periodontal disease, largely influencing the type and patterns of tooth extraction [12]. When tooth loss was studied according to age in Nepal, 80% of extractions because of caries were experienced in people under the age of 40 years, and 70% of periodontal extractions were experienced in people over the age of 40 years [13]. In the United States, over six million people over the age of 50 were edentulous between 2009 and 2014; authors reported that tooth loss was significantly associated with poverty [4].

Considering that the geographical location affects the incidence and, thereby, the trends of tooth extraction, the present study was conducted to study the incidence of tooth loss in Palestine. The aim is to find out which teeth are mostly extracted and to describe the main patterns for this procedure at AQU dental clinics. This investigation would provide valuable insights into the prevalence and reasons for tooth extractions within the Palestinian population, considering the specific context of an educational institution's dental clinic.

## Materials and methods

Patients were approached during their visit to the oral surgery student clinics at AQU during the second semester (from January to May) of the academic year 2021-2022. Consent was obtained by offering patients the opportunity to participate in the study and use their data for research purposes. Patients were informed that participation was voluntary and that their surgical treatment would be unaffected whether they participated or not. The language used to obtain consent was clear and patient-friendly to ensure full understanding. Potential biases in data collection were minimized by ensuring that participation in the study did not impact the standard surgical procedure, thus reducing the likelihood of selection bias.

Inclusion criteria include patients aged between 20 and 80 years old, both genders, both surgical and non-surgical extractions, and all permanent teeth. Exclusion criteria include primary teeth, supernumerary teeth, and uncertain or unclear data.

The sample size was calculated in a simple random sample (for estimating a population proportion) as follows:

Sample size (n) = [(Z^2 * p * (1 - p)) / E^2] Where: n = required sample size; Z = Z-score corresponding to the desired level of confidence (e.g., for a 95% confidence level, Z ≈ 1.96); p = estimated population proportion (if unknown, typically set at 0.5 for maximum variability); E = margin of error (the maximum acceptable difference between the sample estimate and the population parameter).

The surgeon played a critical role in the data collection process. They evaluated the teeth to be extracted and provided final approval for extraction before the procedure. Dental students participated in data collection and tooth extraction, adhering to the regular procedure protocol at the student clinics of AQU. Surgeons and dental students were qualified and trained professionals. Surgeons held the necessary qualifications and expertise for oral surgery. Dental students were under supervision and received appropriate training to ensure consistency in data collection and extraction procedures.

A specific form was filled out for each patient who was going to undergo tooth extraction on the same day. The form underwent pre-testing to ensure clarity and effectiveness in recording the required information. The form contained general patient information, including a dental record of the number of teeth to be extracted on that day, the type of each tooth, and the tooth condition. The evaluation of the tooth to be extracted contained information about the main reasons why the tooth was indicated to be extracted, such as the presence of periodontal attachment loss, history of endodontic treatment, amount of radiographic bone loss, deep carries, and presence of periapical infection. The data collection also asked if this was a clearance case or if the patient had missing teeth due to previous extractions. Also, the number of existing dental implants was recorded. Then, tooth extraction was performed under local anesthesia after the form was filled out by dental students according to the regular procedure protocol at the student clinics of AQU.

Ethical approval for this study was obtained from the Research Ethical Committee at AQU on 27/11/2021 (2021/REC/207). The research data form was only filled out for those patients who signed a written consent. Patients were told that they did not need to accept participation in order to get their surgical treatment performed that day, and that the surgical procedure would be similar, whether they participated or not.

The data was transferred to a Microsoft Excel (Redmond, USA) sheet for analysis by using a specially designed Google Forms (California, USA) sheet for this study. The data were analyzed using IBM Corp. Released 2010. IBM SPSS Statistics for Windows, Version 19.0. Armonk, NY: IBM Corp. The frequency of the variables was calculated as a frequency and percentage.

## Results

After explaining the purposes of the research, none of the attending patients refused to participate in this study. Consent forms were signed by 73 patients attending the AQU student dental clinics for tooth extraction on the day of extraction. The total number of teeth extracted was 151. The participants who underwent extraction were patients who came from the surrounding Palestinian territories.

The youngest patient who underwent permanent tooth extraction was 20 years old, and the oldest patient who underwent extraction was 80 years old. Out of all the participants, 30 patients were female, with an average age of 38.5 years, and 44 were male, with an average age of 48.2 years. For statistical analysis, cases were divided into “non-clearance cases” and “clearance cases”; the latter consisted of patients referred to extract all their remaining teeth, ending up with edentulous jaws. The number of male patients in this study overnumbered the females, and this was repetitive in all groups. The average ages of females were lower in all groups, as shown in Table 1.

**Table 1:** Description of participants according to tooth extraction history and needs; showing differences according to sex groups and with age averages for each group

Variables	Patients who needed extractions	Patients who needed tooth clearance N (%)	Patients who needed non-clearance extractions N (%)	Patients who had no previous extractions N (%)
Number of males	44	11 (25%)	33 (75%)	9 (20.5%)
Average age males (years)	48.2	64.5	42.8	32.1
Number of females	30	4 (13.3%)	26 (86.6%)	7 (23.3%)
Average age females (years)	38.5	54.5	36.0	25.6
Total number of all patients	74	15 (20.3%)	59 (79.7%)	16 (21.6%)
Average age all patients (years)	44.2	61.8	39.8	29.2

The patients were also classified as “first-time” and "not-first-time" according to whether the extraction(s) performed was the first extraction the patient received in their life. This data was obtained by counting the missing teeth and asking the patient about the history of permanent tooth extraction. For the first-time extraction groups, the youngest patient was 20 years old, and the oldest participant was 57 years old. The nine males in this category performed 20.4% in this study, with an average age of 32.1 years. While the seven females performed 23.3% in the study and had an average age of 25.6 years (Table 1). The participants who were “not first-time” to extract had an average age of 48.4 years. The youngest participant in this group is 22 years old, and the oldest is 80 years old. 

Twenty wisdom teeth were extracted in this study. Eleven of them were upper wisdom teeth, and nine were lower wisdom teeth. Out of the 11 upper wisdom teeth, the main reason for extraction was deep caries in nine of them. Deep caries was also the main reason for the extraction of 21 other teeth, distributed equally between different types of teeth. Upper wisdom alone consisted of 30.0% of the teeth extracted due to deep caries. The average age of patients who extracted wisdom teeth with deep caries was 34.6 years. In this study, the remaining roots of 24 teeth were removed. From which, 15 were upper molars and premolars, seven were lower molars and premolars, one was a lower canine, and one was a lower incisor.

Most of the patients came for more than one tooth extraction (62%); only 28 patients came to extract a single tooth. Table 2 shows the main reason for extraction. Results show that caries was the main reason for extraction (62.2% of all attending patients).

**Table 2:** Number of participants according to sex and main reason for tooth extraction

Groups	Males			Females			All
Variables	Non-clearance cases N (%)	Clearance cases N (%)	Total N (%)	Non-clearance cases N (%)	Clearance cases N (%)	Total N (%)	Total N (%)
Caries	20 (60%)	6 (54%)	26 (59.1%)	20 (76.9%)	0 (0%)	20 (66.6%)	46 (62.2%)
Periodontitis	11 (33.3%)	5 (45.4%)	16 (36.4%)	2 (7.6%)	4 (100%)	6 (20.0%)	22 (29.2%)
Impacted wisdom	2 (6.0%)	0 (0%)	2 (4.5%)	4 (15.3%)	0 (0%)	4 (13.3%)	6 (8.1%)
Total	33	11	44	26	4	30	74

The percentage of caries as the main reason was higher in females (66.6%) compared to males (59.1%). However, periodontitis as a main reason for extraction was higher in males (36.4%) than in females (20%), with an overall percentage of 29.2% for all attendees. Patients who came to extract impacted wisdom composed 8.1% of the attendees. In the “non-clearance cases,” the most commonly extracted teeth among the 91 teeth were the lower incisors, followed by the lower first molars. Out of all the teeth extracted in non-clearance cases, 10 (10.9%) were lower incisors, and nine (9.9%) were lower first molars. 

The total number of “clearance cases” included 15 patients. Eleven of them were males and four were females. A total of 60 teeth were extracted in the clearance cases. The most teeth extracted were the lower canines; 13 lower canines were extracted, which consisted of 21.7% of the total teeth extracted in the “clearance” group. On the other hand, only one lower second molar was extracted in this group (1.7%). The lower first molar was not extracted in any of these “clearance cases.” In this study, the average age of the participants for clearance was 61.2 years, with an average of 64.5 years for males and 54.5 years for females. The youngest patient in the “clearance cases” group was a 50-year-old female patient. 

## Discussion

The average age of males (48.2 years) in this study was higher than females (38.5 years); this was recurrent in both clearance and non-clearance groups. This could not specifically reflect the ages of patients attending the student dental clinics at AQU for all various treatments, as such data is not known to the authors. But this may be explained by the fact that females of lower socio-economic conditions may be more concerned about their dental health than males or that younger adult females have a greater willingness to receive treatments at student clinics, which requires more availability of time and more frequent visits to accomplish a certain treatment plan. This is in contrast to private clinics, which would be more efficient in terms of time and speed of treatment. In addition to the fact that student clinics working hours are only in the morning and afternoon, several private clinics provide treatments until the late hours of the day, which might be more convenient for those who work more and have less availability to commit to treatments at student clinics. Another observation was that a greater number of males (59.4%) attended the oral surgery clinics for tooth extraction, but their ages were higher, which may reflect those older males, of whom a good portion may be retired, prefer to attend the student clinics; on the other hand, older females may prefer to attend private clinics and be treated by more experienced dentists. A similar attitude of more males attending a free-service clinic was also reported in Ajman, United Arab Emirates [14].

Results show that caries as the main reason for extraction was significantly higher (62.2%) than any other reason; the second main reason was periodontal disease (29.2%). This may reflect a high incidence of caries among the cohort of patients attending the student clinics at AQU. The incidence of caries as the main reason for extraction was even more obvious in the “non-clearance” cases, which was about three times more than periodontitis as the main reason for extraction. However, “clearance cases” showed a higher incidence of periodontitis as a main reason for extraction. This may reflect the generalized type of periodontal disease. A study done by Takala L et al. [15] explained that clearance cases were of older ages, in which periodontitis could be more severe due to its chronic characteristics [4,15]. Caries has been shown to be the major reason for tooth extraction in other studies [1,2,6,16,17]. 

In this study, the distribution of teeth extracted in “non-clearance cases” showed that the most extracted teeth were upper wisdoms, lower molars, and lower incisors. This may be due to diet habits causing high rates of tooth decay in these teeth. The prevalence of higher numbers of remaining roots in upper molars and premolars may also compensate for the difference, as it was found that there was a need to extract the remaining roots of upper molars and premolars twice as much as lower molars and premolars. However, in a study conducted by Alesia and Khalil [12], it was observed that mandibular anterior teeth were extracted more frequently than maxillary anterior teeth. Remaining roots are a consequence of deep caries, which lead to the destruction of teeth that have not been treated effectively. This may also reflect the fact that lower socio-economic patients would be less able to afford restorative treatments for the teeth that had deep caries, leading to eventual tooth destruction [4,5]. Both high rates of caries and periodontal diseases show that the attending patients have either poor oral health care or that they have less professional dental care, which might be due to both their awareness of oral health and their ability to afford such treatments [14].

For caries as the main reason for extraction, a percentage of 30% were wisdom teeth in this study. This high percentage is due to the fact that usually the first choice of treatment for carious wisdom teeth is extraction due to the difficulty of treatment, especially if root canal treatment is needed [18]. This also reflects the trend among Palestinian dentists, who do not usually advise the extraction of wisdom teeth until problematic conditions arise. Trends vary in different countries about whether to extract asymptomatic disease-free wisdom teeth or to maintain them [19,20]. According to Van der Sanden et al. [21], the variations in decision-making among dental practitioners regarding third-molar treatment can be explained by differences in culture, patient preferences, treatment methods, prevalence of disease, practitioner-to-patient ratio, available resources, reimbursement systems, postgraduate education, and the existence and application of clinical guidelines. The trend in the United States is to extract disease-free wisdom teeth at young ages [22]. A study done by Knutsson K et al. [23] showed a great variation between clinicians when they were asked about their opinion to extract unerupted asymptomatic wisdom teeth; clinicians with more years of experience recommended fewer extractions of non-diseased wisdoms. The overlooking of wisdom tooth extraction in our population may also be due to the expensive cost of extraction of wisdom teeth in the private sector, especially if the extraction needed advanced surgical interventions by a specialist surgeon. The average cost of surgical extraction of a wisdom tooth may range from 150 to 250 USD in an average private clinic in the surrounding Palestinian territories.

The fact that 8.1% of the patients came to AQU clinics to extract their wisdom teeth may reflect that they probably could not afford to cover the expenses of this procedure elsewhere. Another reason may be that they trust to perform the procedure at the AQU clinics with the supervision of a maxillo-facial surgeon rather than to do it at a regular dental clinic [1]. Our results show that the main cause of third-molar extractions was dental caries, which has also been reported to be the main reason for extractions among older adults [5].

Although 78.3% of the attending patients had previous extractions, only one female patient had a dental implant previously placed in her mouth. This can show an inability to afford advanced dental treatment. This could be stated knowing that dental implants are routine dental therapies provided in Palestinian private clinics [24,25], which, unsurprisingly, can be more afforded by patients with higher economic conditions.

The limitations of this study are many, including the fact that it was performed in only one center located in East Jerusalem and provided free treatment to surrounding regions, which may limit the generalizability of the findings to a broader population. Variations in patient demographics, dental practices, and access to healthcare in different regions may not be fully represented, and as mentioned previously, attendees of AQU student university clinics come from a low socio-economic background and are willing to undergo more visits and longer procedures until treatments are accomplished. This narrow cohort is also limited by the number of patients, which would be more representative if performed on all patients attending the clinics for a longer period of at least a whole year. Thus overcoming any seasonal trends of treatment-seeking patients. However, this study is, as far as the authors know, the first study emphasizing this topic in the West Bank, Palestine. The fact that dental treatments are almost exclusively performed in private clinics and the lack of a well-documented health system in Palestine make it very difficult to collect massive amounts of data that can more realistically represent the dental health conditions among Palestinians. The study did not provide a broader context for dental health in Palestine, such as the prevalence of oral diseases, access to dental care, or oral health education programs, which could offer a more comprehensive understanding of tooth extraction trends.

## Conclusions

In conclusion, this study revealed important trends in tooth extraction at the student dental clinic in Palestine. The findings emphasize the significance of addressing dental caries and the extraction of third molars. Based on these results, it is recommended that dental healthcare providers prioritize preventive measures for dental caries and consider strategies for managing third-molar extractions efficiently. Additionally, orthodontic treatment planning should consider the common extraction of lower canines and lower incisors, particularly in clearance cases, to optimize patient outcomes.

To further explore tooth loss prevalence and its determinants among Palestinians, future research should consider longitudinal studies and qualitative investigations. Longitudinal studies could track dental health changes over time, while qualitative research could delve into the socio-cultural factors influencing dental care choices. In light of our findings, it becomes evident that increasing oral health awareness and access to dental care, particularly among individuals with lower socio-economic status, is imperative. Outreach programs, community-based dental education initiatives, and affordable dental services are potential strategies to achieve this goal. These findings also carry potential policy implications. Government-funded dental health programs targeting vulnerable populations and interventions tailored to specific demographics could be instrumental in improving dental care access and reducing the prevalence of dental caries. By expanding our understanding and implementing targeted strategies, we can make substantial strides toward better oral health for all Palestinians.
